# Probing Chromatin Compaction and Its Epigenetic States *in situ* With Single-Molecule Localization-Based Super-Resolution Microscopy

**DOI:** 10.3389/fcell.2021.653077

**Published:** 2021-06-10

**Authors:** Jianquan Xu, Yang Liu

**Affiliations:** ^1^Biomedical Optical Imaging Laboratory, Department of Medicine and Bioengineering, University of Pittsburgh, Pittsburgh, PA, United States; ^2^University of Pittsburgh Hillman Cancer Center, Pittsburgh, PA, United States

**Keywords:** chromatin organization, epigenomics, super-resolution microscopy, single-molecule localization microscopy (SMLM), chromatin dynamics

## Abstract

Chromatin organization play a vital role in gene regulation and genome maintenance in normal biological processes and in response to environmental insults. Disruption of chromatin organization imposes a significant effect on many cellular processes and is often associated with a range of pathological processes such as aging and cancer. Extensive attention has been attracted to understand the structural and functional studies of chromatin architecture. Biochemical assays coupled with the state-of-the-art genomic technologies have been traditionally used to probe chromatin architecture. Recent advances in single molecule localization microscopy (SMLM) open up new opportunities to directly visualize higher-order chromatin architecture, its compaction status and its functional states at nanometer resolution in the intact cells or tissue. In this review, we will first discuss the recent technical advantages and challenges of using SMLM to image chromatin architecture. Next, we will focus on the recent applications of SMLM for structural and functional studies to probe chromatin architecture in key cellular processes. Finally, we will provide our perspectives on the recent development and potential applications of super-resolution imaging of chromatin architecture in improving our understanding in diseases.

## Introduction

**FIGURE 1:**
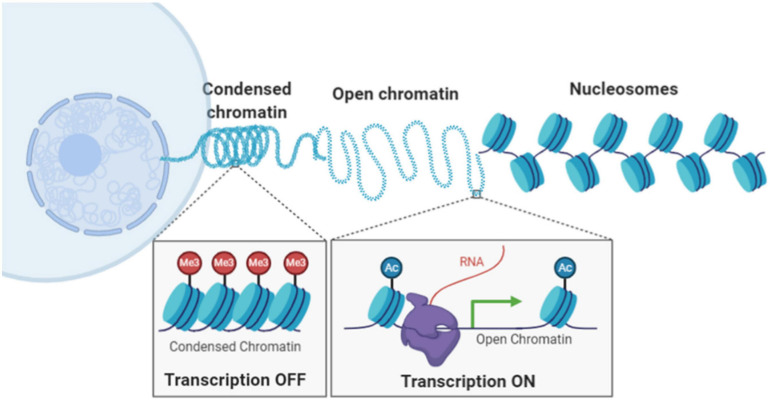
Illustration of hierarchical chromatin organization and different chromatin compaction status inside the cell nucleus.

In eukaryotic cells, chromatin is composed of DNA wrapped around histone proteins, forming the basic building block of nucleosomes assuming “beads-on-a-string” structure. The nucleosomes are further compacted into higher-order chromatin architecture, organized into condensed compartments or heterochromatin domain and open compartments or euchromatin domain (Figure 1). Compaction status of chromatin is in part regulated by chemical modification upon DNA sequences and histone proteins, such as DNA methylation, histone acetylation and methylation. Chromatin compaction regulates transcription activities and impacts many genomic functions such as DNA replication, damage, and repair. Therefore, our ability to probe chromatin architecture and its epigenomic states at molecular scale is essential to our understanding of functional significance of chromatin compaction status and demystify many biological and pathological processes.

Chromatin architecture has been traditionally probed by biochemical assays such as chromatin immunoprecipitation (ChIP) and chromosome conformation capture (3C) based techniques (Lieberman-Aiden et al., 2009), coupled with various molecular biology techniques, next-generation sequencing and bioinformatics analysis. These tools have significantly advanced our understanding of chromatin organization. For example, ChIP-seq analysis helped defining different chromatin states (e.g., promoter, transcribed, repressed) by the enrichment for histone marks at distinct genomic regions (Ernst and Kellis, 2010). Chromatin conformation capture techniques identified the presence of chromatin domains, designated as topologically associating domains (TADs), as the key building blocks of chromatin organization, through genome-scale analysis of contact frequencies between DNA sequences (Dixon et al., 2012, 2016). These biochemical methods remain the mainstream techniques for probing chromatin architecture, but with several limitations. First, they often require a large amount of fragmented DNA sequences from pooled cell population with thousands to millions of cells to generate “averaged” profiles, where single-cell information and spatial context are often lost. Single cell epigenomic profiling is possible (Nagano et al., 2013; Rotem et al., 2015), but it remains a challenging task. Second, their sample preparation protocol is often lengthy and complex, and is sometimes prone to artifacts such as nuclei isolation (Golloshi et al., 2018). Third, these approaches generally work well for isolated cells from cell culture, blood and frozen tissue, but have limited success in formalin-fixed paraffin-embedded tissue (FFPE), the most common form of preservation in preclinical and clinical samples.

Imaging technologies together with quantitative image analysis complement genomic technologies and address many limitations of biochemical methods of probing chromatin architecture. In particular, recent advances in super-resolution fluorescence microscopy revolutionized biological imaging as it surpassed the fundamental diffraction-limited resolution. There are generally three forms of super-resolution fluorescence microscopy techniques including structured illumination microscopy (SIM) (Gustafsson, 2000), stimulated emission depletion microscopy (STED) (Hell and Wichmann, 1994), and single molecule localization microscopy (SMLM) such as (fluorescence) photo-activated localization microscopy [(f)PALM] (Betzig et al., 2006; Hess et al., 2006), (direct) stochastic optical reconstruction microscopy [(*d*)STORM] (Rust et al., 2006; Heilemann et al., 2008) and point accumulation for imaging in nanoscale topography (PAINT) (Sharonov and Hochstrasser, 2006) or DNA-PAINT (Jungmann et al., 2010). We have gained significant insights by directly visualizing chromatin architecture in recent years using conventional high-resolution and super-resolution microscopy (Szabo et al., 2018, 2020; Finn et al., 2019; Miron et al., 2020; Hilbert et al., 2021). Our imaging toolbox for probing chromatin architecture continues to grow and our understanding of chromatin architecture is constantly expanding.

In this short review, we will focus on the application of SMLM-based super-resolution imaging approach in probing chromatin architecture in cells and tissue. SMLM is one of the simplest and cost-effective forms. SMLM can directly visualize chromatin organization and chromatin states at a spatial resolution down to ∼10 nm in single cells even in the spatial context of tissue with limited perturbation to biological samples. Empowered by various fluorescence labeling approach and multiplexing capability, SMLM now emerges as one of the most widely used super-resolution microscopy techniques in *in situ* imaging of nanoscale chromatin architecture such as cultured cells (Ricci et al., 2015; Bintu et al., 2018; Xu et al., 2018), model organisms (Beliveau et al., 2015; Boettiger et al., 2016) and frozen and FFPE tissue (Mateo et al., 2019; Xu et al., 2020). In addition, live-cell imaging based on SMLM and single-particle tracking serves as an important tool to probe dynamic changes of chromatin architecture in live cells (Nozaki et al., 2017; Cho et al., 2018; Xie et al., 2020). Table 1 summarizes the main SMLM techniques and applications discussed in this review.

**TABLE 1:** Summary of main single-molecule localization-based super-resolution microscopy techniques to probe chromatin architecture.

	Labeling strategy	Chromatin targets	Key applications
**STORM**	Antibody-based immunofluorescence	Histone proteins (e.g., core histone or histone proteins)	Global chromatin compaction and its epigenetic states in fixed cells and tissue
	DNA-binding organic fluorophores	Genomic DNA	
	DNA-FISH (Oligonucleotide probes)	Specific DNA sequences	Sequence-specific chromatin imaging, chromatin tracing in fixed cells and tissue
**PALM**	Photoactivatable fluorescent proteins (e.g., Dendra, PA-mCherry, mEos2) or self-labeling tags (e.g., Halo-tag or SNAP-tag) coupled with fluorescent dyes (e.g., Janelia Fluor series)	Genomic DNA and its interaction with DNA regulatory elements	Chromatin dynamic, single molecule tracking in live cells
**ATAC-PALM**	Photoactivatable fluorophore conjugated with Tn5	Accessible chromatin domain	Imaging accessible chromatin and its relationship with DNA regulatory elements in live and fixed cells and tissue
**DNA-PAINT**	Antibody conjugated with docking DNA strand	Histone proteins	Global chromatin compaction in fixed cells
	Oligonucleotide probes conjugated with docking DNA strand	Specific DNA sequences	Sequence-specific chromatin imaging in fixed cells

## Brief Overview of SMLM Imaging System

There have been numerous reviews on SMLM imaging principles and systems in the literature (Patterson et al., 2010; Bates et al., 2013; Deschout et al., 2014; Sage et al., 2015; Sigal et al., 2018). We briefly summarize the key factors and discuss the unique challenges in imaging chromatin architecture. The underlying principle of SMLM is precise localization of single molecules. For the densely labeled structure, a small subset of labeled fluorophores needs to be sequentially turned “on,” whose central positions are localized by mathematical algorithms at nanometer precision. After accumulating localized positions from tens of thousands of imaging frames, the super-resolved image can be reconstructed. It is one of the simplest forms of super-resolution microscope, as it assumes a simple configuration of a wide-field fluorescence microscope coupled with a high-power laser, a high-NA objective (i.e., oil immersion objective with NA > 1.4), an online drift correction module to maintain its nanometer stability during the process of image acquisition. To ensure the best possible resolution, a bright photo-switchable fluorophore is essential coupled with an accurate and robust single molecule localization algorithm.

In principle, imaging chromatin structure in cells and tissue is similar to imaging any other biological structures, except that chromatin is densely packed in the cell nucleus. In addition, the nucleus is several microns thick, located at several microns above the coverslip surface, in comparison to many membrane proteins that are within a few hundred nanometers of the surface. These characteristics presented a few technical challenges. First, the densely packed structure tends to present high and heterogeneous background that can degrade the reconstructed image resolution (Evanko, 2014; Xu et al., 2020). High background mainly comes from unbleached emitters outside the focal plane, which is especially problematic in imaging densely packed chromatin. Total internal reflection (TIRF) configuration is a commonly used method to effectively suppress the background due to its ability to confine light within ∼200 nm above the surface. But for most nuclear targets located at a few microns above, this configuration is not feasible for imaging chromatin in most mammalian cells. To address this issue, lattice light sheet microscopy (Chen et al., 2014) has the ability to generate a ultrathin light sheet of ∼200 nm anywhere inside the cell, effectively suppressing the background. But the instrument is rather complex and requires specialized imaging chamber that is difficult to use. Alternative image processing methods can also be used to mitigate the high background. Spatial filters such as wavelet (Izeddin et al., 2012) or background correction methods (Ma et al., 2019; Ma and Liu, 2020; Mockl et al., 2020) can be used as a pre-processing step prior to single molecule localization to reduce the background noise. On the other hand, emitter sparsity is a requirement for high-quality image reconstruction. For densely packed structure, about 5–10% overlapping emitters may be inevitable. Commonly used sparse emitter localization algorithms can reduce localization accuracy and emitter recall rate, and compromise the image resolution (Holden et al., 2011). Dense emitter localization algorithms are more accurate for localizing overlapping dense emitters (Holden et al., 2011), except that it is computationally intensive and slow (Sage et al., 2019). High-speed algorithms for emitters with moderate or high density (Li et al., 2019; Ma et al., 2019; Xu et al., 2020) have recently become available.

## Super-Resolution Imaging of Chromatin Compaction

**FIGURE 2:**
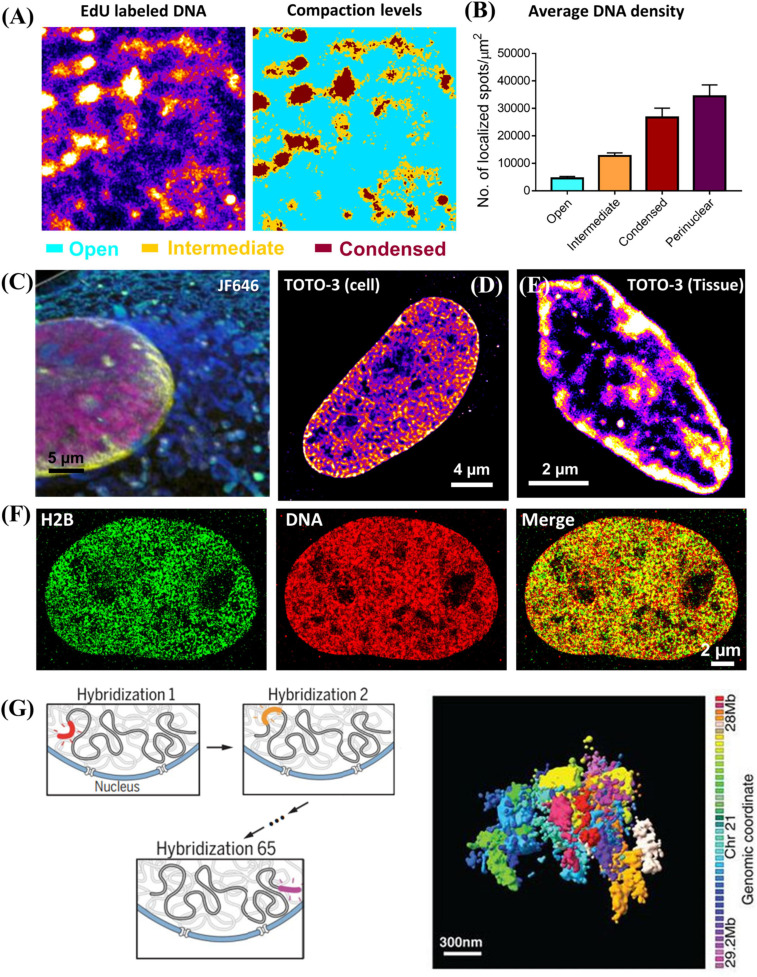
**(A)** Super-resolution image of DNA labeled by EdU conjugated with Alexa 647 and the corresponding three compaction levels (open (cyan), intermediate (brown) and condensed (dark red) chromatin) classified based on k-means clustering. **(B)** The average DNA density (number of localized spots/μm^2^) in open, intermediate, condensed chromatin and nuclear periphery. The error bar is standard error. **(C)** Three-color PAINT imaging of DNA (Hoechst – JF646, PAINT, magenta), nuclear envelope (Dendra 2 – lamin A, PALM, yellow), and intracellular membranes (AzepRh, PAINT, cyan) in a COS-7 cell. This figure is adapted from Legant et al. (2016). **(D,E)** *d*STORM images of TOTO-3 labeled DNA in fixed MCF-10A cell and mouse small intestine tissue. **(F)** Two-color *d*STORM images of H2B (GFP tagged, labeled with CF568 via immunofluorescence staining, Green), and DNA (EdU labeled with Alexa 647, Red) in fixed MCF-10A cell. **(G)** Left: Scheme of multiplexed FISH imaging for high-resolution chromatin tracing for *de novo* identification of chromatin domains. Right: Composite 3D STORM images of 41 consecutive 30-kb chromatin segments in a 1.2-Mb region of chromosome 21 (Chr21:28Mb-29.2Mb), in 41 pseudocolors, in one copy of Chr 21 of an IMR90 cell. This figure is adapted from Bintu et al. (2018).

Chromatin compaction refers to the physical folding of DNA sequences. It was traditionally classified as two simple states: condensed heterochromatin regions and open euchromatin regions. There are three general methods to directly visualize chromatin compaction via super-resolution microscopy, depending on the fluorescently labeled target. The first target is genomic DNA. It does not contain sequence-specific information, and chromatin compaction can be quantified by DNA density reflected in the super-resolution image, with an example shown in Figures 2A,B (Schmid et al., 2017; Xu et al., 2018). Recent evidence has suggested that the higher-order chromatin organization is more complex than a simple dichotomic classification of open vs. condensed states (Cremer et al., 2017). Cellular DNA can be labeled by its precursor analog (e.g., 5-ethynyl-2′-deoxyuridine (EdU), which can be readily incorporated into cellular DNA during DNA replication. Then azide-conjugated dye such as azide-Alexa 647 or azide-CF568 can be added onto EdU-labeled DNA through efficient click chemistry. The main advantages of this approach include its simplicity, a high labeling density and the flexible choice of a wide variety of fluorophores including those best-performing photo-switchable organic fluorophores with high photon number and excellent blinking properties such as Alexa Fluor 647 and CF dyes. But this method labels newly synthesized DNA and the entire genomic DNA is labeled through a full DNA replication cycle. Therefore, it can only be used on live cells or animals, but not on the fixed samples. To visualize genomic DNA in fixed cells or tissue, nucleic acid binding dyes are viable alternatives. For example, Hoechst-JF646 (Grimm et al., 2015; Legant et al., 2016) and TOTO^®^ dye (Xu et al., 2020) have been demonstrated in SMLM imaging of DNA in fixed cells and tissue, as shown in Figures 2C–E, albeit slightly sub-optimal photon number or blinking performance compared to Alexa Fluor 647. Another drawback is that ideally all base pairs should be labeled with the fluorophore, but it often leads to overly high density with unacceptably high background in the densely packed nucleus under the high laser power density needed for STORM. Longer bleaching time and reduced labeling density are often needed to ensure better localization precision and thus high-quality super-resolution image.

The second target is the core histone proteins via immunofluorescence staining. This is also a simple method to visualize global chromatin compaction and the regions of core histone proteins largely overlap with genomic DNA (Figure 2F) and applies to fixed cells and tissue. Based on this approach, Ricci et al. made an important discovery that the nucleosomes form discrete and heterogeneous groups of nanoclusters (or clutches) (Ricci et al., 2015). However, similar to direct labeling of genomic DNA, it does not present any sequence-specific information. Its image quality strongly depends on the quality of the antibody, which sometimes suffers from insufficient labeling density.

The third target is the sequence-specific chromatin imaging via DNA-FISH labeling. The biggest advantage of this approach is that it directly visualizes sequence-specific chromatin compaction at the single cell level. The earlier version of this method targeted the large genomic regions at the scale of a few mega-base pairs. It was later extended to sequential multiplexed STORM or DNA-PAINT imaging to significantly increase the number of targeted sequences, where a set of oligonucleotide probes were used to targeted at each segment with tens of kilobase pairs each round, and the final reconstructed image traces chromatin conformation along the chromosome (Bintu et al., 2018; Nir et al., 2018), as shown in Figure 2G. This approach was further advanced to sequentially label and image an even shorter segment of a few kilobase pairs each round to trace chromatin architecture at a higher resolution with a much faster throughput with automated fluidics control (Mateo et al., 2019). Sequential DNA-FISH combined with barcoding can significantly improve the multiplicity, and enables genome-scale chromatin tracing (∼1000 genomic foci) in the context of landmark nuclear structures (Su et al., 2020). However, they require careful computational design of imaging oligonucleotide probes to minimize non-specific binding to other non-relevant sequences. Such process has been made much easier with the recent development of easy-to-use open-source software packages such as OligoMiner (Beliveau et al., 2018) and ProbeDealer (Hu et al., 2020). However, to visualize a large number of genomic foci, it takes tens of hours to complete imaging tens of thousands of oligonucleotide probes. The active drift-correction module is required to maintain the nanoscale precision throughout the entire imaging time. The automated fluidic system or robotic hands are often needed for washing and adding imaging probes at each round (Boettiger et al., 2016). Therefore, the overall cost is significantly higher than the other techniques discussed above.

## Imaging Chromatin Compaction at Its Epigenetic States

**FIGURE 3:**
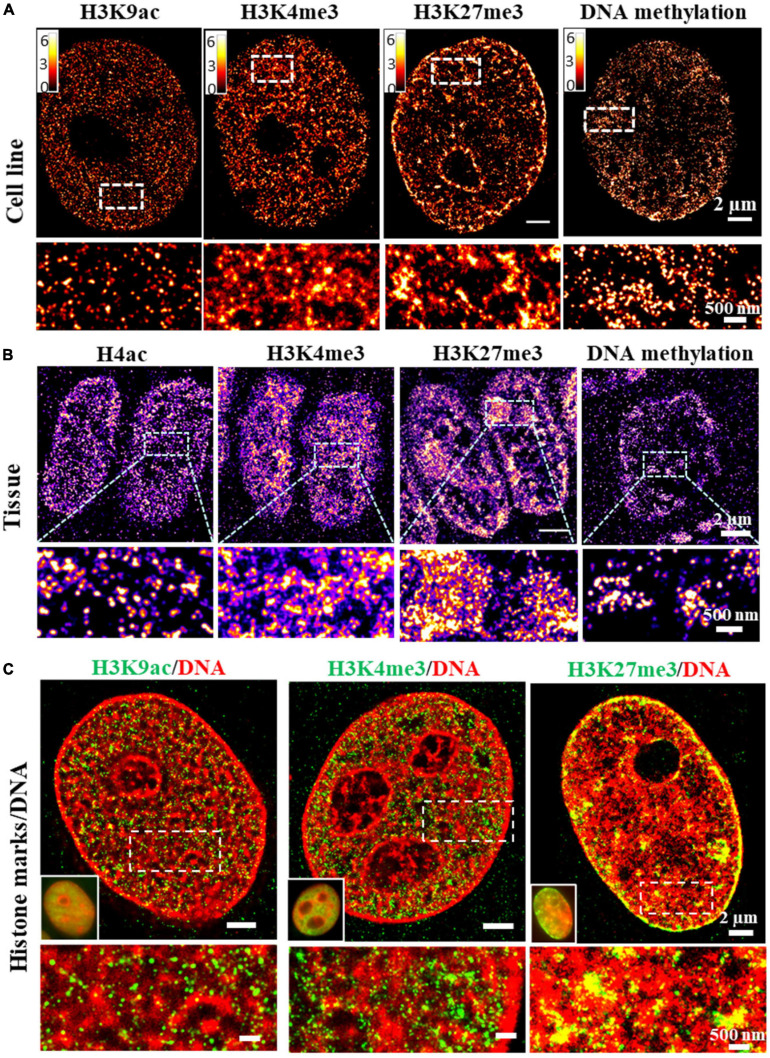
**(A,B)** *d*STORM images of the distinct structures formed by different histone marks and DNA methylation in fixed MCF-10A cells and mouse small intestine tissue. Histone marks and DNA methylation were labeled by immunofluorescence using primary antibodies and Alexa 647 conjugated secondary antibodies. **(C)** Two-color dSTORM images showing the spatial relationship between DNA and different histone marks. Histone marks were labeled by immunofluorescence using primary antibodies and Cy3B conjugated secondary antibodies. DNA was labeled using EdU conjugated with Alexa 647.

Epigenetic states of chromatin are traditionally defined by histone modifications. For example, promoter-associated or active transcription state is enriched by H3K4me3 and H3K9ac, repressed state is enriched by H3K9me3 and H3K27me3 (Ernst and Kellis, 2010) and enhancer region is enriched by H3K4me1 and H3K27ac. The compaction status of chromatin can be indirectly derived from the enrichment of these histone proteins at specific genomic regions from ChIP-seq based assays. The increased H3K4me3 or H3K9ac proteins generally suggest more open chromatin, while increased H3K27me3 or H3K9me3 suggest more condensed chromatin. However, the bulk protein level is not a direct reflection of the physical compaction of chromatin folding. Super-resolution imaging of these histone proteins or other epigenetic factors in conjunction with genomic DNA can serve as a simple approach to directly visualize chromatin compaction at its epigenetic states at the genome scale. Super-resolution imaging of histone proteins enriched at distinct epigenetic states in cultured mammalian cells and tissue has revealed different compaction structures. For example, histone acetylation (e.g., H3K9ac) forms mostly spatially segregated nanoclusters, active histone methylation (e.g., H3K4me3) forms mostly spatially dispersed nanodomains, while repressed histone methylation forms mostly large and compact aggregates [Figures 3A,B, modified from our previous work (Xu et al., 2018, 2020)]. Similarly, the global structure of methylated DNA can also be visualized in single cells by fluorescently labeling 5-methylcytosine (5 mC) (from our unpublished data). As shown in Figures 3A,B, clustered methylated DNAs showed more accumulation at nuclear periphery and some regions in the nucleus similar to the pattern in heterochromatin. When overlaid with genomic DNA, spatial relationship between DNA compaction and its adjacent histone proteins can be revealed. For example, at the genomic scale, promoter-associated active histone mark H3K4me3 was found to largely overlap with open DNA regions, while repressed histone mark H3K9me3 or H3K27me3 was found to overlap with most dense regions of DNA (Xu et al., 2018), as shown in Figure 3C. The advantage of this approach is its simplicity and the ability to visualize spatial organization of any epigenetic factor that can be labeled via immunofluorescence staining. However, it does not provide any sequence-specific information. However, it should be noted that immunofluorescence-based labeling for SMLM often involves a target-specific primary antibody and a detection secondary antibody conjugated with fluorophore for signal amplification. The large size of the antibody (∼7 nm) can introduce a displacement between the fluorophore reporter and the actual target, referred to as “linkage error” (Ries et al., 2012).

Single molecule localization microscopy-based super-resolution imaging can also be used to selectively target accessible chromatin by tagging a photoactivatable fluorophore with Tn5 (Xie et al., 2020). As this labeling strategy is built upon a similar principle as ATAC-seq (Buenrostro et al., 2013), it is referred to as ATAC-PALM. It is a convenient method to study accessible chromatin and can be applied to live and fixed cells as well as tissue. It uniquely targets accessible chromatin domains which have been shown to be statistically co-localize with active (H3K4me3-enriched) genomic regions. ATAC-PALM recently revealed that the nuclear YAP (a transcriptional co-activator) condensates localize to spatially segregated accessible chromatin domains, which become sites for active gene transcription (Cai et al., 2019).

To visualize sequence-specific chromatin states, the targeted sequence enriched in a specific or a combination of histone marks or important regulatory elements can first be identified from ChIP-seq data. These targeted sequences can then be labeled using DNA-FISH discussed above and imaged using SMLM (Boettiger et al., 2016). This method seamlessly connects genomic data or traditional approaches of defining epigenetic states with the super-resolution imaging at the single cell level. Chromatin compaction and its epigenetic states can be visualized in the context of the conventional knowledge gained from epigenetic studies. However, beyond a limited number of targeted sequences, imaging genome-scale chromatin states can be very costly and time-consuming.

## Imaging Chromatin Dynamics in Live Cells

Understanding chromatin architecture is not just limited to visualizing spatial arrangement of DNA sequences or chromatin compaction, its dynamic behavior is also an essential component. Chromatin dynamics reflects local chromatin compaction to some extent, informs the interaction of chromatin with neighboring environment, and fluid or solid like chromatin behavior (Zidovska, 2020). Conventional live-cell fluorescence imaging has been widely used to study chromatin dynamics, such as fluorescence recovery after photobleaching (Strom et al., 2017) and single particle tracking. In recent years, SMLM-based super-resolution imaging has been increasingly used with single-particle tracking to investigate chromatin dynamics at nanoscale resolution.

**FIGURE 4:**
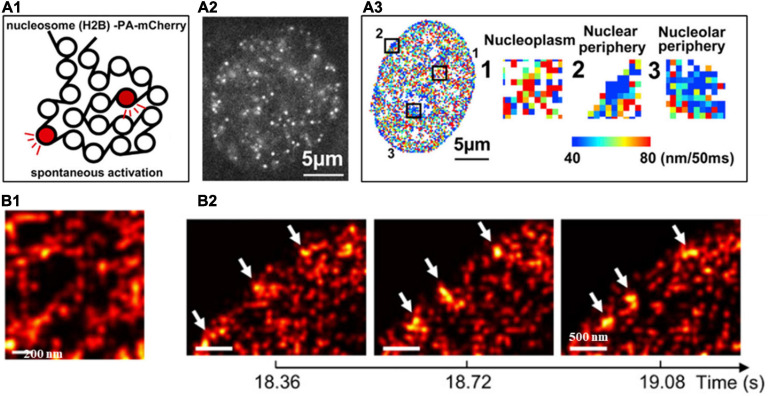
**(A1)** Scheme of histone H2B-PA-mCherry activation for live PALM imaging and single-nucleosome tracking. **(A2)** Single-nucleosome (H2B-PA-mCherry) image of the nucleus of a live HeLa cell. **(A3)** The chromatin heatmap for 50 milliseconds in a live HeLa cell and magnified images from the boxed regions in the heatmap. **(B1)** Super-resolution images of segregated accumulations of H2B within a chromatin-rich region in a live cell nucleus. **(B2)** Three consecutive super-resolution images showing the dynamic structure (arrows) in a live cell nucleus. These figures are adapted from Nozaki et al. (2017); Barth et al. (2020).

The target (e.g., core histone, RNA polymerase II, transcription factors) in the nucleus can be fluorescently labeled with genetically encoded proteins by fusing a photoconvertible (or photoactivatable) fluorescent protein such as Dendra, PA-mCherry, or mEos2) (Cisse et al., 2013) or self-labeling tags (SNAP-tag, or Halo-tag) coupled with organic fluorophores such as Janelia Fluor series (Grimm et al., 2015). Photoconversion or photoactivation creates sparse distribution of fluorescent emitters, which can be used for localization and its motion can be followed for a short period of time (milliseconds to seconds). This PALM-based super-resolution imaging combined with single particle tracking were first applied to characterize the spatiotemporal organization the transient clustering of RNA polymerase II (RNAPII) in live eukaryotic cells with single-molecule sensitivity (Cisse et al., 2013). This approach was later applied to image the dynamics of single nucleosomes to infer chromatin architecture and local chromatin compaction. For example, PALM-based single nucleosome imaging in live cells revealed that nucleosomes form compact nanodomains with reduced movement in heterochromatin-rich regions (Nozaki et al., 2017), through the multi-distance spatial clustering analysis (Figure 4A). The similar approach has also been used to extract its interaction with neighboring environment that RNAPII globally constraints chromatin movement (Nagashima et al., 2019) via the analysis of mean square distance of nucleosomes upon inhibition of RNAPII. More recently, high-density PALM combined with deep learning was applied in live cells to inform both structural and dynamic properties of chromatin architecture (Barth et al., 2020). This approach identified the elongated chromatin domain “blobs” at sub-100-nm range as well as the highly dynamic behavior of chromatin blobs that usually form and dissemble within less than 1 s (Figure 4B).

On the other hand, PALM-based super-resolution imaging has also been used to understand the dynamics of DNA-binding transcription factors and chromatin environment. For example, the combination of PALM imaging with lattice light sheet microscopy was used to investigate enhancer organization in live embryonic stem cells and the transcription factor target search process (Liu et al., 2014). The similar imaging approach has also been applied to show the association between mediator and RNAPII clusters with chromatin that behaved as phase-separated condensates (Cho et al., 2018). Both studies used lattice light sheet microscopy, which extended the capability of traditional 2D imaging to fast 3D imaging and tracking of transcription factors in the nucleus. Due to the minimized photobleaching, the tracking time of the target was also extended from seconds to even hours. Understanding these functionally important dynamic behaviors of chromatin architecture and its relationship in regulatory elements will continue to be an important subject in biological research.

## Super-Resolution Imaging of Chromatin Architecture in Biological Processes

Besides the investigation of fundamental chromatin architecture and its dynamic behaviors in fundamental cell and molecular biology, SMLM-based super-resolution imaging of chromatin architecture has been slowly demonstrating its application to understand other normal and pathological processes. Two of the noticeable areas are developmental biology and cancer.

In developmental biology, there has been well-established evidence that stem cells assume an open chromatin state that is crucial to maintenance of pluripotency (Gaspar-Maia et al., 2011). Understanding chromatin architecture in stem cells at nanoscale is a natural fit for SMLM as one of its earliest applications. For example, PALM imaging and lattice light sheet microscopy revealed the relationship between enhancer-binding pluripotency regulator (Sox2) and chromatin organization in live stem cells (Liu et al., 2014). Sox2 enhancers form 3D clusters that are spatially segregated from heterochromatin but largely overlapped with RNAPII-enriched regions, which also likely impacts the 3D-diffusion dominant Sox2 target search mode. Such study opens the opportunity for the continued effort to understand how 3D organization of enhancer clustering influences gene activity.

**FIGURE 5:**
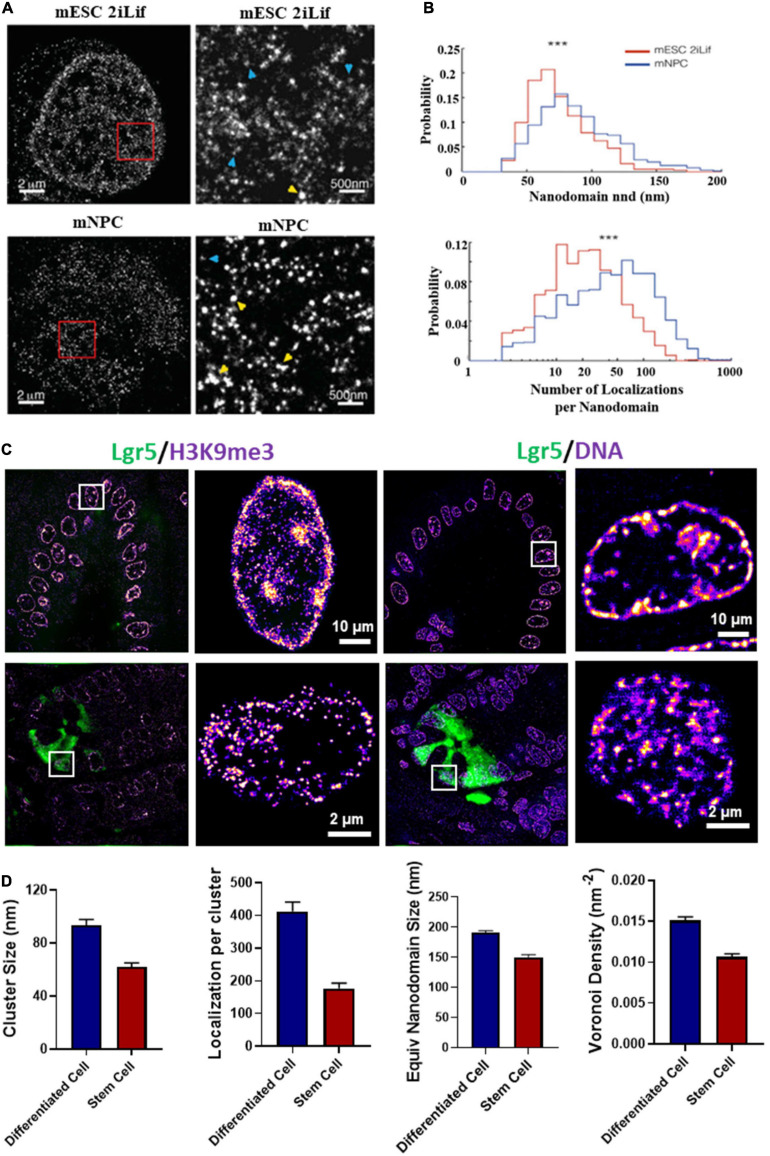
**(A)** dSTORM images of H2B in type 1 mouse embryonic stem cells (mESCs) cultured in 2iLif and neuronal precursor cells (mNPC) obtained after differentiation of mESCs. This figure is adapted from Ricci et al. (2015). **(B)** Representative distributions of the number of H2B localizations per nanodomain and nanodomain nearest neighbor distance (nnd) in mESCs cultured in 2iLif medium (red) and mNPCs (blue) for the cells shown in **(A)**. **(C)** Chromatin structure marked by H3K9me3 and DNA of intestinal stem cells (Lgr5-EGFP marked green color) located at the crypt regions and differentiated cells located at the villi regions from a EGFP-labeled Lgr5 (Lgr5-EGFP-Ires-CreERT2) mouse. Lgr5 marks the intestinal stem cells. **(D)** Quantification of H3K9me3 and DNA nanodomain size (or cluster size) and the number of localized molecules per cluster for the cells in **(C)**. DNA nanodomain size and local density of DNA quantified by Voronoi polygon density between differentiated intestinal epithelial cells in the villi and intestinal stem cells (Lgr5 +) in the crypts. About 50–100 cells were analyzed for each group. *P*-values were calculated using Mann-Whitney test.

Besides live-cell imaging, STORM imaging revealed that pluripotent stem cells contained less dense nucleosome nanoclusters (referred to as clutches) compared to somatic cells and the clutch size strongly correlated with pluripotency potential of induced pluripotent stem (iPS) cells (Ricci et al., 2015; Figures 5A,B). This important discovery not only provided the direct imaging evidence at the single cell level that supports the well-accepted “open” chromatin state in pluripotent stem cells, but also suggested the possibility of using nanoscale clutch size to measure pluripotency potential in iPS cells. Beside iPS cells, our group showed that the intestinal stem cells exhibited more open chromatin or significantly smaller nanodomains formed by heterochromatin or dense regions of DNA (Figures 5C,D) compared to differentiated cells (from our unpublished data).

**FIGURE 6:**
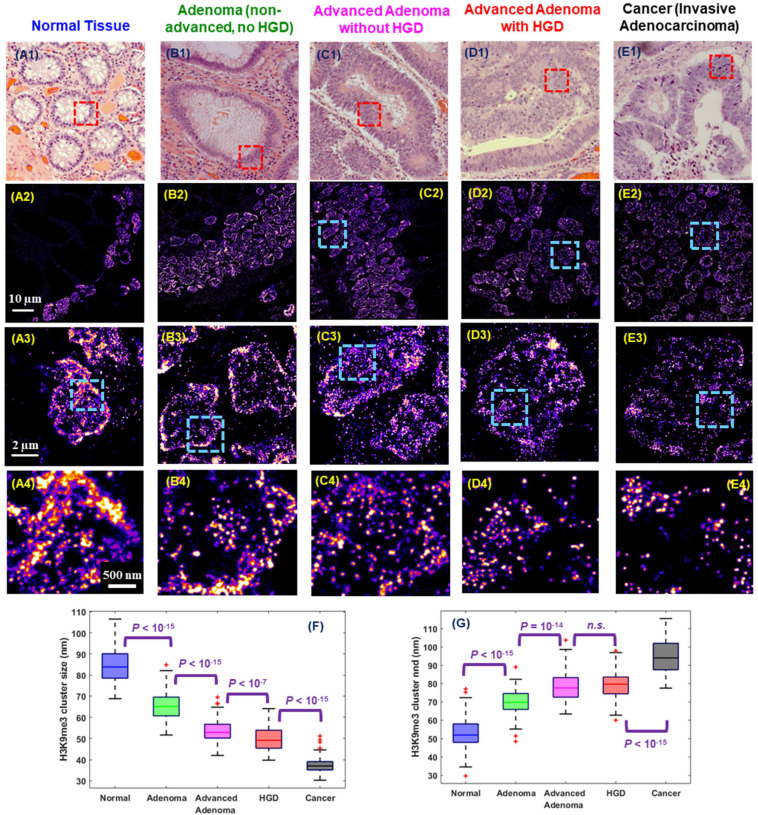
Representative **(A1–E1)** histology and **(A2–E2)** corresponding super-resolution images of nanoscale H3K9me3-dependent heterochromatin structures (from the red boxes) in normal tissue and tumors representing progressive stages of colorectal carcinogenesis including adenoma, advanced adenoma without high-grade dysplasia (HGD), advanced adenoma with HGD, and invasive adenocarcinoma from human colon tissue. Note that adenoma and advanced adenoma without HGD are histologically indistinguishable, and advanced adenoma without HGD refers to those large adenomas with a tumor size of more than 1 cm (1.5 cm polyps). **(A3–E3)** and **(A4–E4)** are the progressively zoomed images of **(A2–E2)**. **(F,G)** The box-and-whisker plots of the cluster size and nearest neighbor distance (nnd) of H3K9me3-dependent heterochromatin. About 100–200 nuclei analyzed for each group. *P* values were determined using Mann-Whitney test.

Interestingly, cancer cells bear certain resemblance to stem cells given their ability to divide perpetually to support tumor growth. Similarly, their higher-order chromatin conformation also shares some similarities. Indeed, abnormal chromatin structure is one of the most universal and striking characteristics in cancer cells and remains the gold standard for cancer diagnosis for two centuries (Nickerson, 1998; Zink et al., 2004). The established abnormal chromatin structure in cancer cells includes many microscopic features such as enlarged nuclear size, irregular shape and coarse chromatin texture (Zink et al., 2004; Fischer et al., 2010). At the molecular scale, numerous studies supported epigenetic dysregulation in carcinogenesis such as aberrant DNA methylation and histone modification in carcinogenesis (McDonald et al., 2011; Sandoval and Esteller, 2012; Reddy and Feinberg, 2013; Feinberg et al., 2016). However, the manifestation of the dysregulated epigenome as molecular-scale higher-order chromatin structure in carcinogenesis were previously not well defined in the preserved spatial context of pathological tissue (Reddy and Feinberg, 2013). Xu et al. (2020) optimized SMLM imaging protocol to FFPE tissue and uncovered a gradual de-compacted and fragmented higher-order chromatin structure at all stages of carcinogenesis in multiple tumor types in the mouse models. Such gradually disrupted heterochromatin structure was also observed at different stages of human tumor and Figure 6 shows an example of progressive stages of colon carcinogenesis from normal cells to precursors (adenoma and high-grade dysplasia) to invasive cancer. This study also demonstrated the potential of SMLM imaging of nanoscale chromatin disruption to identify the high-risk precursors that cannot be distinguished by conventional pathology, such as adenoma and advanced adenoma without high-grade dysplasia, suggesting its potential for risk stratification. Although it remains at its infancy, SMLM imaging of chromatin architecture – at nanoscale resolution – may open a new avenue for improving cancer diagnosis or facilitating the future development and evaluation of new early detection strategies, beyond conventional microscopic evaluation of morphology, while complementing the fast-growing genomic technologies in cancer research.

Besides imaging chromatin architecture in tumor cells, direct visualization of chromatin compaction has also been shown in the activation of immune cells. Kieffer-Kwon et al. (2017) recently reported that stimulated lymphocytes decondense chromatin that showed spreading and decompaction of histone nanodomains. Such de-compacted chromatin architecture reduced transcription factor residence time and provided accessibility to DNA binding regulatory proteins (Kieffer-Kwon et al., 2017). In these process, Myc oncogene served as a master regulator of chromatin architecture in B cell activation. This study underlies the importance for future investigation of chromatin architecture at the interface between immunology and oncology.

## Limitations of SMLM

Despite the simplicity and superior resolution of SMLM, we also recognize its limitations. SMLM technique is based on the precise localization of central positions of sparsely distributed individual fluorescent emitters, followed by accumulation of localized points from thousands of imaging frames. Such imaging mechanism requires photo-switching (“blinking”) and high photon number from the fluorophore for optimal image quality and resolution. Unfortunately, only a handful of fluorophores present such properties such as Alexa 647 (or structural analog Cy5) (Dempsey et al., 2011), a few CF dyes (Diekmann et al., 2020) and photo-switching fluorescent proteins (Wang et al., 2014). Further, photo-switchable dyes require specific imaging conditions (e.g., high laser power, presence of oxygen scavenger, primary thiol environment), which are not well suited for live cell imaging. PAINT or DNA-PAINT mitigates the limitations of using photo-switchable dyes through transient binding of any fluorophore to the imaging target to generate stochastic blinking events. But it requires a much longer time to accumulate sufficient sampling for image reconstruction and the diffusing fluorophores generate high background.

## Perspectives

The field of super-resolution microscopy and SMLM have undergone the most exciting technical revolution in the past 15 years. Most technical bottlenecks have been addressed, from high-speed image reconstruction algorithms to high throughput and automation in imaging system, to the applications of various quantitative image analysis. The SMLM has become faster, more robust, and cheaper. These technical advancements coupled with numerous innovations in fluorescence labeling strategies of *in situ* hybridization and fluorescent probes have truly revolutionized our ability to learn about chromatin architecture at nanometer resolution over thousands of cells in the spatial context of tissue architecture, pathological status and nuclear landmarks. We envision that the field will rapidly shift the focus from technical development and proof-of-concept phase to the application phase that focuses on addressing important questions in biological systems and unmet clinical needs in patient care. We expect that super-resolution imaging will be increasingly used as a routine tool to visualize molecular-scale chromatin architecture at the single-cell level, which will be integrated with genomic and transcriptomic data with the assistance of powerful bioinformatics tools to gain a comprehensive understanding of complex normal and pathological processes. Super-resolution imaging of chromatin architecture may also lead to new diagnostic tests.

## Author Contributions

YL and JX: conceptualization and writing. YL: supervision. Both authors discussed the results and commented on the manuscript.

## Conflict of Interest

The authors declare that the research was conducted in the absence of any commercial or financial relationships that could be construed as a potential conflict of interest.
